# Finite element analysis of cross fluid model over a vertical disk suspended to a tetra hybrid nanoparticles mixture

**DOI:** 10.1038/s41598-024-51262-w

**Published:** 2024-01-17

**Authors:** Muhammad Sohail, Umar Nazir, Abha Singh, Ayele Tulu, Muhammad Jahangir Khan

**Affiliations:** 1 Institute of Mathematics, Khwaja Fareed University of Engineering and Information Technology, Rahim Yar Khan, 64200 Pakistan; 2 Department of Mathematics, Faculty of Science, Khon Kaen University, Khon Kaen, 40002 Thailand; 3 Department of Basic Sciences, College of Sciences and Theoretical Studies, Dammam-Branch, Saudi Electronic University, Riyadh, Saudi Arabia; 4 Department of Mathematics, CNCS Ambo University, Ambo, Ethiopia; 5 Department of Advance Materials and Technologies, Faculty of Materials Engineering, Silesian University of Technology, 44-100 Gliwice, Poland

**Keywords:** Energy science and technology, Engineering, Materials science, Mathematics and computing, Nanoscience and technology, Physics

## Abstract

Nanoparticles have numerous applications and are used frequently in different cooling, heating, treatment of cancer cells and manufacturing processes. The current investigation covers the utilization of tetra hybrid nanofluid (aluminum oxide, iron dioxide, titanium dioxide and copper) for Crossflow model over a vertical disk by considering the shape effects (bricks, cylindrical and platelet) of nanoparticles, electro-magneto-hydrodynamic effect and quadratic thermal radiation. In the current inspection model is first derived given PD-equations and then altered into a system of OD-equations by including similarity variables. The converted ordinary differential equations are solved by using the finite element procedure and the impact of the solution against numerous involved parameters is displayed through tables and graphs. It is observed that tetra-hybrid nanoparticles are recommended better in industrial applications where the highest production of thermal energy. Moreover, an enhancement of thermal production can be achieved utilizing different values of the magnetic parameter, time relaxation number, variable thermal radiation number and magnetic induction number but the opposite trend has been noticed with the effects of radiation number.

## Introduction

Understanding the idea of heat is crucial in distinct engineering domains. Heat transmission is fundamental particularly to civil as well as mechanical and chemical engineers on account of significant impacts on material choice, equipment effectiveness, and reaction kinetics, respectively. Many fields utilize heat transfer techniques, including automotive engineering and thermal proficiency of electronic devices as well as systems, climate control, chemical engineering, insulation, materials mechanism and power plant engineering. In heat transportation, Nanofluids are generally employed as coolant equipment including heat exchangers, electronic cooling systems (such as flat plates), and radiators due to their improved thermal characteristics. Several scholars have studied heat transmission on flat plates. As a power function of the displacement from the slot, the stretching velocity changes. Thermal radiation and viscous dissipation are included in the conservation of energy equation to assist the mechanical activities of the heat transfer mechanism. Configured by a moving vertical plate enclosed by a penetrating surface, a comparison of pure fluid (water) and nanofluid (Cu–H_2_O) is explored. The work is innovative in that it uses an incompressible fluid in an unstable laminar MHD natural transmission flow to achieve the thermal conductivity specifically of nanofluid that is greater than that of pure fluid. Furthermore, the chemical response of this specific nanofluid under consideration of radiation absorption is detected by taking into account the nanoparticle's ability to reach thermic equilibrium is discussed by Arulmozhi et al. ^1^. One of the most typical phenomena in biological systems is peristaltic. The impacts of curvature, porosity, rheology, and heat transmission often play a role in certain bodily systems, particularly the digestive, reproductive, respiratory, and renal systems. 

As a result, in the current study, we combine the Sisko fluid’s flow through a porous media enclosed by curving, wavelike walls with the phenomena of heat transmission. As a consequence of vasomotion (peristaltic motion) within the artery, the theoretical approach offered under the long wavelength approximation behaves as a model in response to the creeping non-isothermal flow specifically of blood through a sick part referring to the artery examined by Asghar et al. ^2^. Benos et al. ^3^ expressed that a horizontal wall resembling a sponge and enabling mass transpiration is used in examining heat transportation in the circumstances of sheets stretching as well as contracting. In-depth research is done on the radiation impacts, and the external magnetic field as well as under consideration of the Prandtl number. Bilal et al. ^4^ investigated how the iron oxide traditionally water-based along with CNT hybrid nanofluid flow amongst the binary spinning plates when magnetic and electro-hydrodynamic influences are coupled. The primary objective of this study is to determine how electrically MHD affects mass as well as heat transfer features. The laminar magneto-hydrodynamic flow occurrence (MHD flow) modeled for upper-convected Maxwell fluid bounded by an isothermal penetrating extended surface has been reported by Guled et al. ^5^ with the aid of the optimum homotropy analysis (OHAM) methodology. The findings of research on the influences of variables such the relaxation time, generation/absorption velocity as well and magnetic number in response to the velocity across a sheet are compared to the comparable results that were previously accessible. This article discusses how the MHD free stream's nanofluid flow and heat transmission across an exponentially radiated stretched sheet interact with both constant and variable fluid properties. Irfan et al. ^6^ explained the rate appertaining to mass and heat transportation in MHD specifically based on Williamson nanofluid flow bounded by exponentially penetrable elongating surface that is susceptible to mass suction and heat generation/suction. Li et al. ^7^ convey that additionally, a magnetic field from the outside is supplied at an oblique angle along the stretched surface. Malik et al. ^8^ indicate that a constant, laminar boundary layer flow of Sisko fluid to a melting stretched surface of the same material. An adequate transformation converts the modeled partial differential equations into ordinary differential equations. This research seeks to examine the application of the projection-based embedded discrete fracture model framework based on the numerical modeling of dual-phase heat and mass transportation in fractured reservoirs described by Rao et al. ^9^. Projection-based embedded discrete fracture model (PEDFM) is a recently developed numerical simulation framework for mass transportation subjected to fractured reservoirs. The manufacturing of biological polymers, medicines, and other environmentally acceptable purposes all make use of bio-convection. Since the heat transportation rate towards the extending surface dictates the final product quality, many fundamental applications, including the fabrication of plastic films and also polymer sheets. Moreover, it relies on extending surface technology. As a result, Saranya et al. ^10^ investigate the bio-convective heat transportation brought on by gyrotactic microbes swimming within a nanofluid across an unstable curved extending sheet. The dual diffusion theory referring to a model based on heat flux has been quantitatively investigated by Ali et al. ^11^ for the finite element analysis of Maxwell’s transient magneto-hydrodynamic rotating flow in three dimensions and tangent hyperbolic-shaped nanofluidic flow across a bidirectional elongating sheet. The flow-regulating boundary layer equations take into consideration the effects of thermophoresis along with Brownian movement. In the three-dimensional flow occurrence specifically of the Sisko fluid, the effects appertaining to the magnetic field and nanoparticles are modeled in this article. A surface that can extend both ways is what is causing the flow. Brownian movement along with thermophoresis effects are under consideration while developing the nanofluid model. By Hayat et al. ^12^ it is believed that Sisko fluid electrically conducts via an applied magnetic field that is always present. The primary goal of the current investigation is to better understand the thixotropic nanofluid’s magneto-hydrodynamic (MHD) nonlinear convective flow. A nonlinear extending surface with varying thickness is what causes flow. The energy expression takes into account nonlinear thermal radiation along with heat generation/suction. Zero mass flux within the sheet and convective circumstances are taken into consideration. Hayat et al. ^13^ research idea revolves around the creation of a model for nanomaterials that incorporates thermophoresis and Brownian motion phenomena. Here, a full discussion of the characteristics of the heat transportation mechanism and the impact of the relaxation parameter referring to the current viscous nanofluid flow is provided by Khan et al. ^14^. In a square container that has been partly heated and is filled with nanofluids, this article discusses how buoyancy force enhances heat transmission. When square-shaped horizontal walls are in motion towards opposing directions to one another, the model is created by Kumar et al. ^15^ to report the behavior referring to nanofluids based on volume fraction and stretching factors. In industries, the use of nano-fluids is extremely important. The GO-MoS_2_ nanocomposite is used in energy storage and water purification and has notable photocatalytic activity. In this issue, Nayak et al. ^16^ have examined nonlinear thermal radiation as well as heat absorption. Moreover, viscous dissipation towards the three-dimensional GO-MoS_2_/Casson hybrid nanofluidic flow occurrence amongst the dual parallel plates. To understand the dynamics referring to the Casson hybrid nanofluid bounded by a bidirectional nonlinear elongating sheet, this work will examine the roles of mixed convection along with Brownian movement and thermophoresis impacts. Combining Tiwari, Das, and Buongiorno’s models is taken into account for the flow model. Puneeth et al. ^17^ expressed that the descriptive flow equations appearing in the Casson hybrid nanofluid model are transformed into a system based on independent variables with the aid of suitable similarity transformation. Jena et al. ^18^ studied the uneven flow of tiny fluid across a porous vertical layer caused by the collision of a disposed magnetic field. Pattnaik et al. ^19^ examined the independent convection of an electrically conductive.

The literature review reveals that there is no study related to tera-Cross nanofluid in heat transfer mechanism over a vertical disk with following gaps.

- The rheology of Cross nanofluid is studied on vertical disk;
- Four kinds of nanoparticles aluminum oxide, copper, titanium oxide and iron dioxide are inserted;
- Induced magnetic field and electric field are studied;
- Joule heating and variable thermal radiations are addressed;
- Non-Fourier’s theory is utilized for the characterization of the heat transfer mechanism;
- Thermal conductivity and viscosity models (bricks, cylindrical and platelet) for hybrid nanoparticles are utilized;
- A finite element method is implemented for finding complex model.

It is claimed that the above-mentioned points illustrate that there is no work exists in published articles ^41–44^. A system related to PDEs is transformed into a system of ODEs using similarity variables. The obtained system of ODEs is mathematically resolved with the help of the finite element approach. Heat transfer and flow mechanisms have been formulated and discussed in the next sections.

## Mathematical analysis

**Figure 1:**
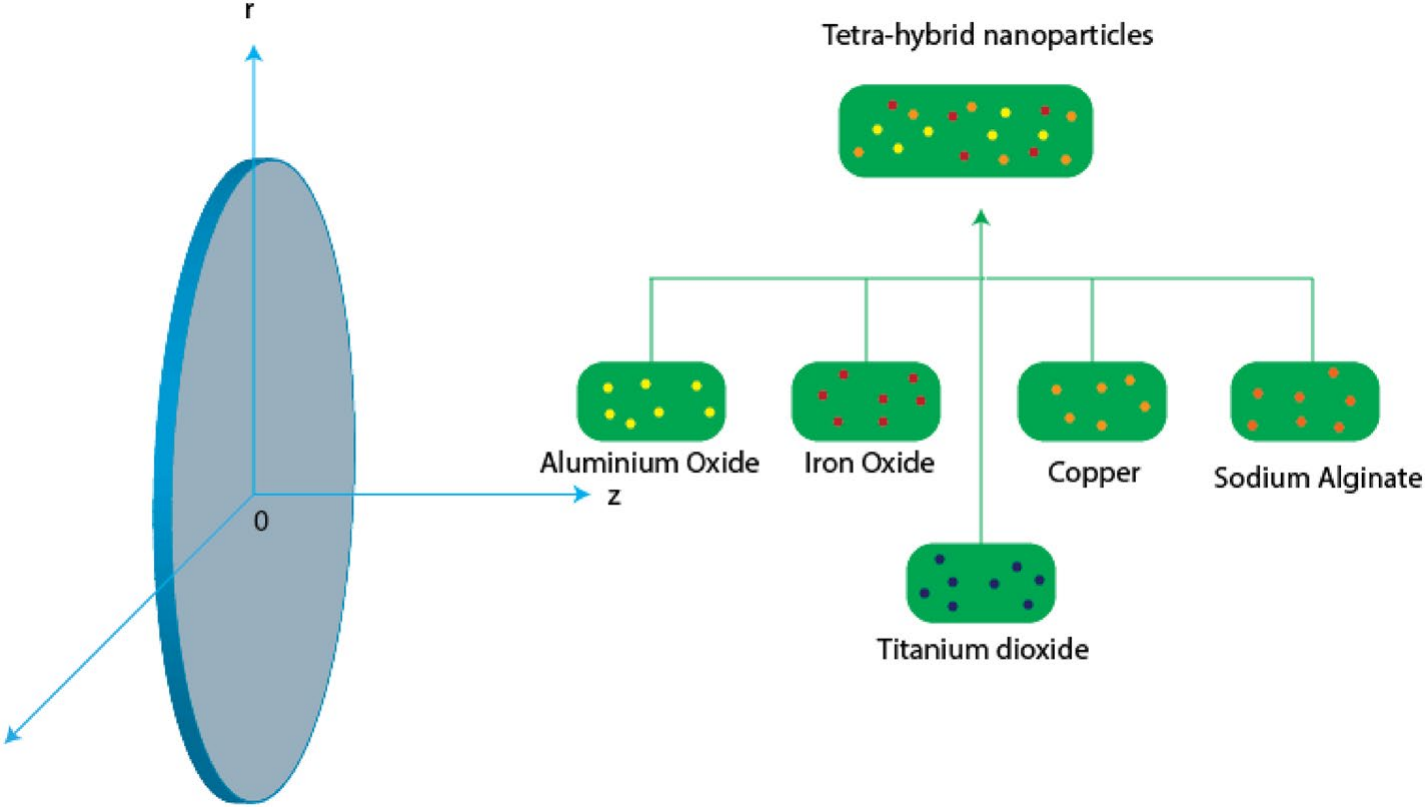
Schematic flow model.

Here, two-dimensional, steady and viscous cross-rheology across a vertical disk utilizing the second law regarding thermal analysis. Recent developments related to tetra hybrid nanofluid have implemented unitizing-based fluid (Sodium alginate). Nanofluids are based on aluminum oxide, iron dioxide, titanium dioxide and copper. The motion of tetra-hybrid nanoparticles is produced using wall velocity ${U}_{w}\left(=ar\right)$ and the surface regarding disk is convectively heated through wall temperature ${T}_{w}$ whereas tetra-hybrid nanoparticles are flowing over the surface with region $z=0.$ An induced magnetic field and electrical field are implemented past a surface. The energy equation contains quadratic and linear thermal radiation with a non-Fourier approach. Thermal properties based on tetra-hybrid nanoparticles in a base fluid are addressed in Table 1 while Fig. 1 shows a schematic model of the current model. The constitutive governing flow ^41^ model implements boundary layer approximations that are defined as

**Table 1:** Thermal properties of $\sigma , k$, $\rho$ and Sodium alginate ^47,48^.

Nano-particles	$k$	$\rho$	$\sigma$
${Al}_{2}{O}_{3}$	32.9	6310	$5.96\times {10}^{7}$
${Fe}_{3}{O}_{4}$	80	5180	$0.112\times {10}^{6}$
$Cu$	401	8933	$59.5\times {10}^{6}$
Sodium alginate	0.6376	989	$5.01\times {10}^{-6}$
$Ti{O}_{2}$	8.9538	686.2	4250

1 $${U}_{r}+\frac{U}{r}+{W}_{Z}=0,$$

$$U{U}_{r}+W{U}_{z}-\frac{\ddot{\mu }}{\pi 4{\rho }_{Tetra}}\left({H}_{1}{\left({H}_{1}\right)}_{r}+{H}_{3}{\left({H}_{1}\right)}_{Z}\right)=-\frac{\ddot{\mu }}{\pi 4{\rho }_{Tetra}}{H}_{E}\frac{d{H}_{E}}{dr}+{\left(\frac{\sigma }{\rho }\right)}_{Tetra}E{B}_{0}$$

2 $$\begin{array}{c}-{\nu }_{Tetra}{\left[1+{\Gamma }^{n}{\left({U}_{Z}\right)}^{n}\right]}^{-1}-n{\nu }_{Tetra}{\left[1+{\Gamma }^{n}{\left({U}_{Z}\right)}^{n}\right]}^{-2}{\Gamma }^{n}{\left({U}_{Z}\right)}^{n}-{\left(\frac{\sigma }{\rho }\right)}_{Tetra}{\left({B}_{0}\right)}^{2}U, \end{array}$$

3 $$\begin{array}{c}{\left({H}_{1}\right)}_{r}+\frac{{H}_{1}}{r}+{\left({H}_{3}\right)}_{Z}=0, \end{array}$$

$$U{T}_{r}+W{T}_{Z}+{\delta }_{*}\left(\begin{array}{c}{U}^{2}{T}_{rr}+2UW{T}_{rZ}+{W}^{2}{T}_{ZZ}+U{W}_{r}{T}_{Z}\\ +U{U}_{r}{T}_{r}+W{U}_{Z}{T}_{r}+W{W}_{Z}{T}_{Z}\end{array}\right)$$

4 $$\begin{array}{c}=\frac{{k}_{Tetra}}{{\left(\rho {C}_{p}\right)}_{Tetra}}{T}_{ZZ}+\frac{16{\sigma }^{*}}{{\left(\rho {C}_{p}\right)}_{Tetra}3{k}^{*}}\left({T}^{3}{T}_{ZZ}+3{T}^{2}{\left({T}_{Z}\right)}^{2}\right). \end{array}$$

The physical constraints are imposed ^41^ as

5 $$\begin{array}{c}\left.\begin{array}{c}u=ar, T={T}_{W}, W=0, {\left({H}_{A}\right)}_{z}=0, {H}_{B}={H}_{C}=0 as Z=0\\ \to {u}_{\infty }, W\to 0, T\to {T}_{\infty },{H}_{A}\to {H}_{0}r when z\to \infty \end{array}\right\}. \end{array}$$

For obtaining ODEs imposing following transformations ^41^

6 $$\begin{array}{c}u=ar{F}_{\eta }, W=-2{\left(a{\nu }_{f}\right)}^\frac{1}{2}F, \eta =Z{\left(\frac{a}{{\nu }_{f}}\right)}^\frac{1}{2}, {H}_{A}={H}_{0}r{G}_{\eta }, {H}_{C}=-2{\left(a{\nu }_{f}\right)}^\frac{1}{2}G, \Theta =\frac{T-{T}_{\infty }}{{T}_{w}-{T}_{\infty }}. \end{array}$$

Using Eq. (6), the foremost PDEs ^41^ are

7 $$\begin{array}{c}\left.\begin{array}{c}\left(1+\left(1-n\right){We}^{n}{\left({F}_{\eta \eta }\right)}^{n}\right){\left(1+{We}^{n}{\left({F}_{\eta \eta }\right)}^{n}\right)}^{-2}{F}_{\eta \eta \eta }-\frac{{\nu }_{f}}{{\nu }_{Tetra}}\left[\begin{array}{c}{\left({F}_{\eta }\right)}^{2}-2F{F}_{\eta \eta }\\ -\beta \left({\left({G}_{\eta }\right)}^{2}-2G{G}_{\eta \eta }-1\right)\end{array}\right]\\ -\frac{{\sigma }_{f}}{{\sigma }_{Tetra}}{M}^{2}{F}_{\eta }+\frac{{\sigma }_{f}}{{\sigma }_{Tetra}} E^{*}  {M}^{2}=0,\\ {F}_{\eta }\left(0\right)=1,{F}_{\eta }\left(\infty \right)\to A, F\left(0\right)=0,\end{array}\right\}, \end{array}$$

8 $$\begin{array}{c}\left.\begin{array}{c}\lambda {G}_{\eta \eta \eta }+2F{G}_{\eta \eta }-2{F}_{\eta \eta }G=0,\\ {G}_{\eta \eta }\left(0\right)=1, {G}_{\eta }\left(\infty \right)\to 1,G\left(0\right)=0 \end{array}\right\}, \end{array}$$

9 $$ \left. {\begin{array}{*{20}l}    {\Theta _{{\eta \eta }}  + \frac{{k_{f} \left( {\rho C_{p} } \right)_{{Tetra}} }}{{k_{{Tetra}} \left( {\rho C_{p} } \right)_{f} }}PrF\Theta _{\eta }  - \frac{{k_{f} \left( {\rho C_{p} } \right)_{{Tetra}} }}{{k_{{Tetra}} \left( {\rho C_{p} } \right)_{f} }}Pr\Omega _{a} \left[ {FF_{\eta } \Theta _{\eta }  + F^{2} \Theta _{\eta } } \right]} \hfill  \\    { + Rd\left[ \begin{gathered}   3\left( {\Theta _{w}  - 1} \right)\left( {\Theta _{\eta } ^{2}  + \Theta ^{2} \Theta _{\eta } ^{2} \left( {\Theta _{w}  - 1} \right)^{2}  + 2\Theta \Theta _{\eta } ^{2} \left( {\Theta _{w}  - 1} \right)} \right) + \Theta _{{\eta \eta }}  + \Theta ^{3} \Theta _{{\eta \eta }} \left( {\Theta _{w}  - 1} \right)^{3}  \hfill \\    + 3\left( {\Theta _{w}  - 1} \right)\Theta \Theta _{{\eta \eta }}  + 3\left( {\Theta _{w}  - 1} \right)^{2} \Theta ^{2} \Theta _{{\eta \eta }}  \hfill \\   \Theta \left( 0 \right) = 1,\Theta \left( \infty  \right) \to 0. \hfill \\  \end{gathered}  \right] = 0} \hfill  \\   \end{array} } \right\}. $$

Correlations for tetra-hybrid nanofluid ^45^ are defined as

10 $$\begin{array}{c}{\mu }_{Tetra}={\mu }_{f}{\left[{\left(1-{\ell}_{4}\right)}^{2.5}{\left(1-{\ell}_{3}\right)}^{2.5}{\left(1-{\ell}_{2}\right)}^{2.5}{\left(1-{\ell}_{1}\right)}^{2.5}\right]}^{-1}, \end{array}$$

11 $${\rho }_{Tetra}=\left[\left(1-{\ell}_{4}\right)\left\{\left(1-{\ell}_{3}\right)\left(1-{\ell}_{2}\right)\left(\left(\left(1-{\ell}_{1}\right)+\frac{{\ell}_{1}{\rho }_{s1}}{{\rho }_{f}}\right)\right)+\frac{{\ell}_{2}{\rho }_{s2}}{{\rho }_{f}}+\frac{{\ell}_{3}{\rho }_{s2}}{{\rho }_{f}}+\frac{{\ell}_{4}{\rho }_{s2}}{{\rho }_{f}}\right\}\right],$$

12 $${\left(\rho {C}_{p}\right)}_{Tetra}=\left[\begin{array}{c}\left(1-{\ell}_{4}\right)\left(1-{\ell}_{3}\right)\left(1-{\ell}_{2}\right)\left(1-{\ell}_{1}\right)\left(\left(1-{\ell}_{1}\right)+\frac{{\ell}_{1}{\left(\rho {C}_{p}\right)}_{s1}}{{\left(\rho {C}_{p}\right)}_{f}}\right)+\frac{{\ell}_{2}{\left(\rho {C}_{p}\right)}_{s2}}{{\left(\rho {C}_{p}\right)}_{f}}\\ +\frac{{\ell}_{3}{\left(\rho {C}_{p}\right)}_{s3}}{{\left(\rho {C}_{p}\right)}_{f}}+\frac{{\ell}_{4}{\left(\rho {C}_{p}\right)}_{s4}}{{\left(\rho {C}_{p}\right)}_{f}}\end{array}\right],$$

13 $$\begin{array}{c}\frac{{k}_{Tetra}}{{k}_{f}}=\frac{\left({k}_{s4}+2{k}_{tri}-2{\ell}_{4}\left({k}_{tri}-{k}_{s4}\right)\right)}{\left({k}_{s4}+2{k}_{tri}+{\ell}_{4}\left({k}_{tri}-{k}_{s4}\right)\right)}, \frac{{k}_{Tri}}{{k}_{f}}=\frac{\left({k}_{s3}+2{k}_{hy}-2{\ell}_{3}\left({k}_{hy}-{k}_{s3}\right)\right)}{\left({k}_{s3}+2{k}_{hy}+{\ell}_{3}\left({k}_{hy}-{k}_{s3}\right)\right)}, \end{array}$$

14 $$\begin{array}{c}\frac{{k}_{hy}}{{k}_{f}}=\frac{\left({k}_{s2}+2{k}_{Nf}-2{\ell}_{2}\left({k}_{Nf}-{k}_{s2}\right)\right)}{\left({k}_{s2}+2{k}_{Nf}+{\ell}_{2}\left({k}_{Nf}-{k}_{s2}\right)\right)}, \frac{{k}_{Nf}}{{k}_{f}}=\frac{\left({k}_{s1}+2{k}_{f}-2{\ell}_{1}\left({k}_{f}-{k}_{s1}\right)\right)}{\left({k}_{s1}+2{k}_{hy}+{\ell}_{1}\left({k}_{f}-{k}_{s1}\right)\right)}, \end{array}$$

15 $$\begin{array}{c}\frac{{\sigma }_{Tetra}}{{\sigma }_{f}}=\frac{\left({\sigma }_{s4}+2{\sigma }_{tri}-2{\ell}_{4}\left({\sigma }_{tri}-{\sigma }_{s4}\right)\right)}{\left({\sigma }_{s4}+2{\sigma }_{tri}+2{\ell}_{4}\left({\sigma }_{tri}-{\sigma }_{s4}\right)\right)}, \frac{{\sigma }_{Tri}}{{\sigma }_{f}}=\frac{\left({\sigma }_{s3}+2{\sigma }_{hy}-2{\ell}_{3}\left({\sigma }_{hy}-{\sigma }_{s3}\right)\right)}{\left({\sigma }_{s3}+2{\sigma }_{hy}+2{\ell}_{3}\left({\sigma }_{hy}-{\sigma }_{s3}\right)\right)}, \end{array}$$

16 $$\begin{array}{c}\frac{{\sigma }_{hy}}{{\sigma }_{f}}=\frac{\left({\sigma }_{s2}+2{\sigma }_{Nf}-2{\ell}_{2}\left({\sigma }_{Nf}-{\sigma }_{s2}\right)\right)}{\left({\sigma }_{s2}+2{\sigma }_{Nf}+2{\ell}_{2}\left({\sigma }_{Nf}-{\sigma }_{s2}\right)\right)}, \frac{{\sigma }_{Nf}}{{\sigma }_{f}}=\frac{\left({\sigma }_{s1}+2{\sigma }_{f}-2{\ell}_{1}\left({\sigma }_{f}-{\sigma }_{s1}\right)\right)}{\left({\sigma }_{s1}+2{\sigma }_{f}+2{\ell}_{1}\left({\sigma }_{f}-{\sigma }_{s1}\right)\right)}, \end{array}$$

17 $$\frac{{k}_{tetra}}{{k}_{f}}=\left[\begin{array}{c}\frac{\left({k}_{s4}+2{k}_{tri}-2{\ell}_{4}\left({k}_{tri}-{k}_{s4}\right)\right)}{\left({k}_{s4}+2{k}_{tri}+{\ell}_{4}\left({k}_{tri}-{k}_{s4}\right)\right)}*\frac{\left({k}_{s3}+2{k}_{hy}-2{\ell}_{3}\left({k}_{hy}-{k}_{s3}\right)\right)}{\left({k}_{s3}+2{k}_{hy}+{\ell}_{3}\left({k}_{hy}-{k}_{s3}\right)\right)}\\ \frac{\left({k}_{s2}+2{k}_{hy}-2{\ell}_{2}\left({k}_{Nf}-{k}_{s2}\right)\right)}{\left({k}_{s2}+2{k}_{hy}+{\ell}_{2}\left({k}_{hy}-{k}_{s2}\right)\right)}*\frac{\left({k}_{s1}+2{k}_{f}-2{\ell}_{1}\left({k}_{Nf}-{k}_{s1}\right)\right)}{\left({k}_{s1}+2{k}_{f}+{\ell}_{1}\left({k}_{f}-{k}_{s1}\right)\right)}\end{array}\right] ,$$

18 $$\begin{array}{c}\frac{{k}_{tetra}}{{k}_{f}}=\left[\begin{array}{c}\frac{\left({\sigma }_{s4}+2{\sigma }_{tri}-2{\ell}_{4}\left({\sigma }_{tri}-{\sigma }_{s4}\right)\right)}{\left({\sigma }_{s3}+2{\sigma }_{tri}+{\ell}_{4}\left({\sigma }_{tri}-{\sigma }_{s4}\right)\right)}*\frac{\left({\sigma }_{s3}+2{\sigma }_{hy}-2{\ell}_{3}\left({\sigma }_{hy}-{\sigma }_{s3}\right)\right)}{\left({\sigma }_{s3}+2{\sigma }_{hy}+{\ell}_{3}\left({\sigma }_{hy}-{\sigma }_{s3}\right)\right)}\\ \frac{\left({\sigma }_{s2}+2{\sigma }_{Nf}-2{\ell}_{2}\left({\sigma }_{Nf}-{\sigma }_{s3}\right)\right)}{\left({\sigma }_{s3}+2{\sigma }_{Nf}+{\ell}_{3}\left({\sigma }_{Nf}-{\sigma }_{s3}\right)\right)}*\frac{\left({\sigma }_{s1}+2{\sigma }_{f}-2{\ell}_{1}\left({\sigma }_{f}-{\sigma }_{s1}\right)\right)}{\left({\sigma }_{s1}+2{\sigma }_{f}+{\ell}_{1}\left({\sigma }_{f}-{\sigma }_{s1}\right)\right)}\end{array}\right]. \end{array}$$

**Figure 2:**
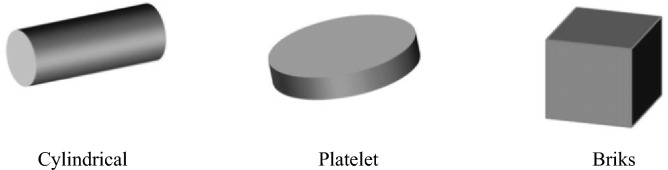
Diagrams of different shapes of nanoparticles.

Viscosity and thermal conductivity models ^46^ (bricks, cylindrical and platelet) for hybrid nanoparticles are defined as follows and their sketch is shown in Fig. 2

19 $$\begin{array}{c}\frac{{\mu }_{Nf2}}{{\mu }_{bf}}=1+904.4{\ell}^{2}+13.5l, {K}_{nf1}={K}_{bf}\left[\frac{{K}_{s{p}_{2}}+3.9{K}_{bf}-3.9\ell\left({K}_{bf}-{K}_{s{p}_{2}}\right)}{{K}_{s{p}_{2}}+3.9{K}_{bf}+\ell\left({K}_{bf}-{K}_{s{p}_{2}}\right)}\right], \end{array}$$

20 $$\begin{array}{c}\frac{{\mu }_{Nf3}}{{\mu }_{bf}}=1+612.6{\ell}^{2}+37.1l, {K}_{nf1}={K}_{bf}\left[\frac{{K}_{s{p}_{3}}+4.7{K}_{bf}-4.7\ell\left({K}_{bf}-{K}_{s{p}_{3}}\right)}{{K}_{s{p}_{3}}+4.7{K}_{bf}+\ell\left({K}_{bf}-{K}_{s{p}_{3}}\right)}\right], \end{array}$$

21 $$\begin{array}{c}\frac{{\mu }_{Nf1}}{{\mu }_{bf}}=1+471.4{\ell}^{2}+1.9l, {K}_{nf1}={K}_{bf}\left[\frac{{K}_{s{p}_{1}}+0.81{K}_{bf}-0.81\ell\left({K}_{bf}-{K}_{s{p}_{1}}\right)}{{K}_{s{p}_{1}}+0.81{K}_{bf}+\ell\left({K}_{bf}-{K}_{s{p}_{1}}\right)}\right]. \end{array}$$

Weissenberg number, magnetic parameter, electric field number, magnetic field parameter, Prandtl number, radiation number, temperature difference number, magnetic Prandtl number and time relaxation number are defined as

$$We=\Gamma a{Re}^{1/2}, \beta =\frac{\ddot{\mu }}{\pi 4{\rho }_{f}}{\left(\frac{{H}_{0}}{a}\right)}^{2}, E^{*}  =\frac{{E}_{0}}{{B}_{0}{\left({C}_{p}\right)}_{f}}, M=\frac{{{\sigma }_{f}\left({B}_{0}\right)}^{2}}{a{\rho }_{f}},$$

$$Pr=\frac{{\left(\mu {C}_{p}\right)}_{f}}{{k}_{f}}, Rd=\frac{4{\sigma }_{f}{T}_{\infty }^{3}}{{k}_{f}{k}^{*}}, {\Theta }_{w}=\frac{{T}_{w}}{{T}_{\infty }}, {\lambda =\frac{{\mu }_{E}}{{\nu }_{f}} ,\Omega }_{a}={\delta }_{*}a.$$

Aluminum oxide has high stability and thermal conductivity that is visualized as remarkable applicants for enhancing the thermal performance and thermal assets of fluids. Iron oxide has magnetic properties that are useful in various applications related to magnetism. Copper exhibits excellent electrical conductivity that is useful in electronics and conductive applications associated with hybrid procedures. Titanium dioxide is utilized in photocatalytic properties and titanium dioxide is known for applications including catalysis. Preparing a mixture of aluminum oxide, iron oxide, copper and titanium dioxide with Cross fluid has properties that are useful in electrical conductivity, enhancing thermal conductivity, magnetic responsiveness, photocatalysis and hybrid systems. The thermal properties of aluminum oxide, iron oxide, copper and titanium dioxide are mentioned in Table 1.

Skin friction coefficient ^41^ is defined as

22 $$\begin{array}{c}{C}_{f}=\frac{{\tau }_{w}}{{\rho }_{f}{\left({U}_{w}\right)}^{2}}, \sqrt{{R}_{e}}{C}_{f}=\left(\frac{1}{{\left(1-{\ell}_{4}\right)}^{2.5}{\left(1-{\ell}_{3}\right)}^{2.5}{\left(1-{\ell}_{2}\right)}^{2.5}{\left(1-{\ell}_{1}\right)}^{2.5}}\right)\frac{{F}^{{\prime}{\prime}}\left(0\right)}{1+{\left(We{F}^{{\prime}{\prime}}\left(0\right)\right)}^{2}}. \end{array}$$

Divergent of temperature ^41^ (Nusselt number) is defined as

23 $$\begin{array}{c}Nu=\frac{-r\left({k}_{Tetra}+\frac{16{T}_{\infty }^{3}{\sigma }^{*}}{3{k}^{*}}\right){T}_{Z}{|}_{Z=0}}{{\left({T}_{w}-{T}_{\infty }\right)k}_{f }},{\left({R}_{e}\right)}^{-\frac{1}{2}}Nu=-\frac{{k}_{Tetra}}{{k}_{f}}{\left(1+Rd{\left({\Theta }_{w}\right)}^{3}\right)\Theta }_{\eta }\left(0\right), \end{array}$$

where (Reynolds number) $Re\left(=\frac{r{U}_{w}}{{\nu }_{f}}\right).$

## Finite element method

The present model in the form of ODEs is numerically resolved by a finite element scheme while the assigned domain is discretized into small segments named as approach. The utilization of approach in chemical processing, electrical systems, computational problems and solid mechanics. Steps related to the FE-approach are listed below.

*Step 1*: In this step, the defined strong form is transformed into weak forms and residuals are generated;

*Step 2*: Linear kind shape functions are considered and weak forms are achieved via Galerkin finite element;

*Step 3*: Global stiffness matrix and stiffness elements are achieved by implementing the assembly process;

*Step 4*: The concept of Picard linearization has been used for obtaining a system regarding linear equations;

*Step 5*: Computational tolerance is assumed as ${10}^{-5}$ for algebraic equations and the stopping range is defined as

24 $$\begin{array}{c}\left|\frac{{\varsigma }_{i+1}-{\varsigma }_{i}}{{\varsigma }^{i}}\right|<{10}^{-5}. \end{array}$$

*Step 6*: Convergence analysis (mesh-free analysis) is assumed by Table 2.

**Table 2:** Analysis of grid size of ${F}_{\eta }\left(\frac{{\eta }_{max}}{2}\right), {G}_{\eta }\left(\frac{{\eta }_{max}}{2}\right)$ and $\Theta \left(\frac{{\eta }_{max}}{2}\right)$.

$e$	${F}_{\eta }\left(\frac{{\eta }_{max}}{2}\right)$	${G}_{\eta }\left(\frac{{\eta }_{max}}{2}\right)$	$\Theta \left(\frac{{\eta }_{max}}{2}\right)$
30	0.006810274801	0.1138430976	0.00008343370242
60	0.005228358273	0.1072571830	0.00004885290704
90	0.004754609121	0.1051132127	0.00004025378789
120	0.004592643069	0.1106967323	0.08045189988
150	0.004458081446	0.1100395457	0.07911715040
180	0.004369756846	0.1096032761	0.07823435560
210	0.004307338243	0.1092925964	0.07760718150
240	0.004260883946	0.07713854572	0.1090600859
270	0.004224964012	0.1088795157	0.07677512057
300	0.004196357048	0.1087352985	0.07648506098

### Validation of current work

Table 2 illustrates the validation of the current investigation given Skin friction coefficient with published work ^41^. Various values of $\beta =0.0, 0.5, and 1.0$ are recorded in Table 2. It was found that a good comparative simulation has been noticed. Present work is simulated by FEM while published work is simulated Optimal HAM approach. Moreover, FEM associated code is designed on MAPLE 18. Table 2 addresses a mesh-free study for 300 elements.

## Discussion and results

A vertical disk is considered for visualizations of complex fluid with suspensions of different kinds of nanoparticles. Utilizations of various shape impacts based on cylindrical, bricks and platelets are addressed by implementing a non-Fourier approach. A finite element scheme is the most helpful approach for obtaining such a complex model. Tetra-hybrid correlations are implemented for the investigation of thermal performance. Here, detailed studies related to temperature profile and motion due to nanoparticles are displayed versus different parameters.

**Figure 3:**
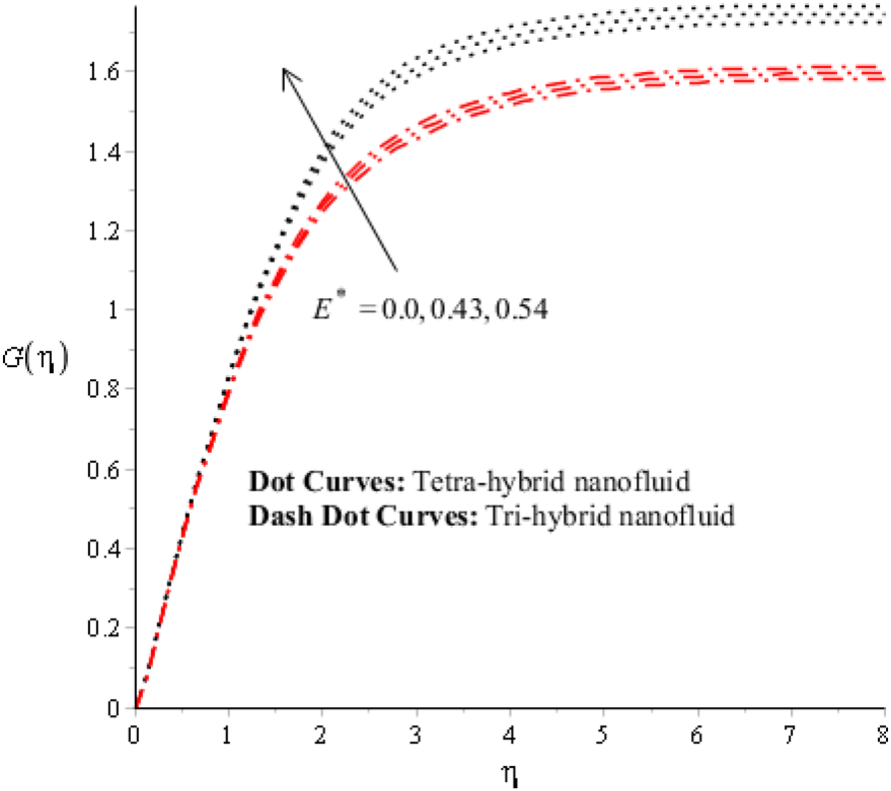
Impression of $E^{*}$ on $G\left(\eta \right).$

**Figure 4:**
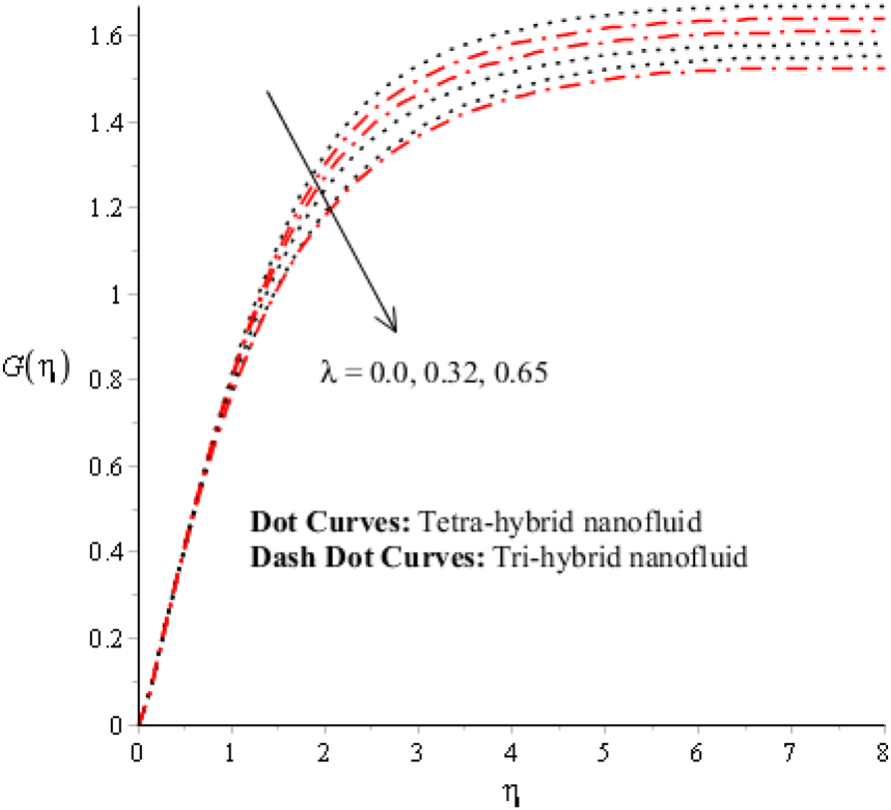
Impression of $\lambda$ on $G\left(\eta \right)$.

**Figure 5:**
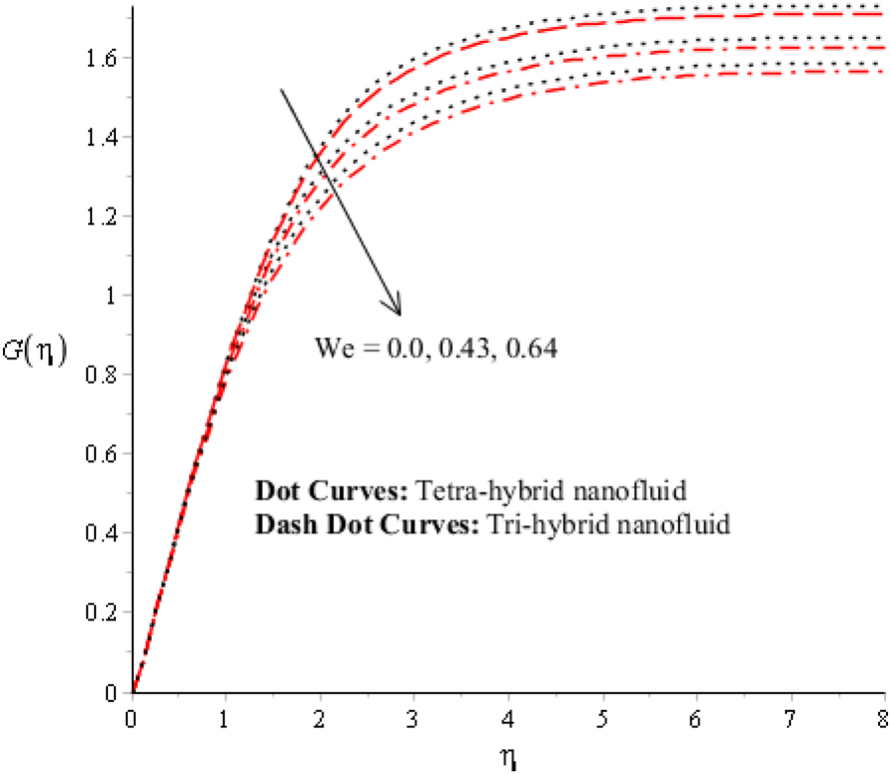
Impression of $We$ on $G\left(\eta \right).$

**Figure 6:**
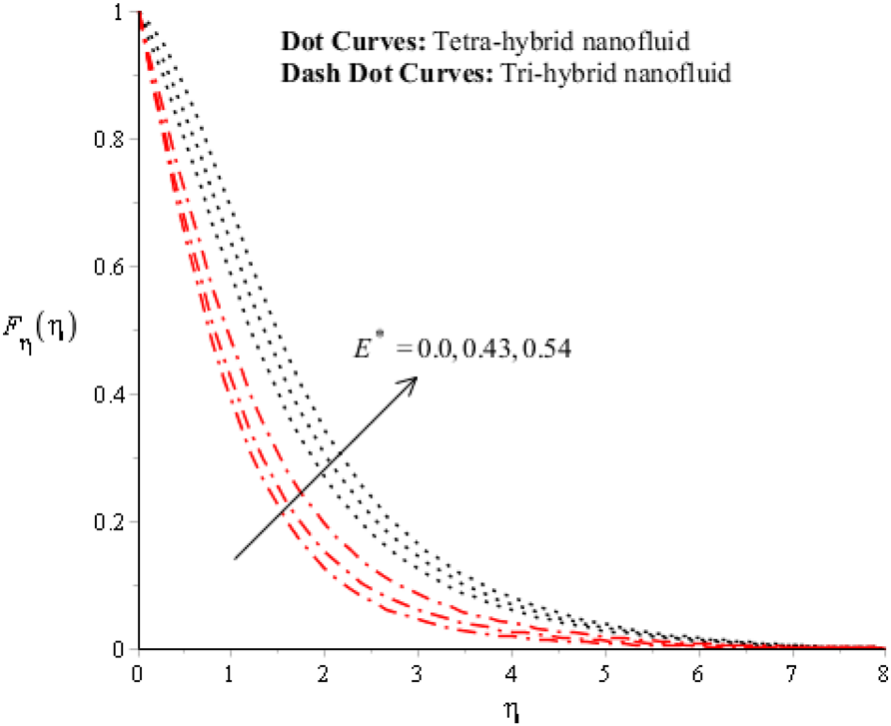
Impression of $E^{*}$ on ${F}_{\eta }\left(\eta \right).$

**Figure 7:**
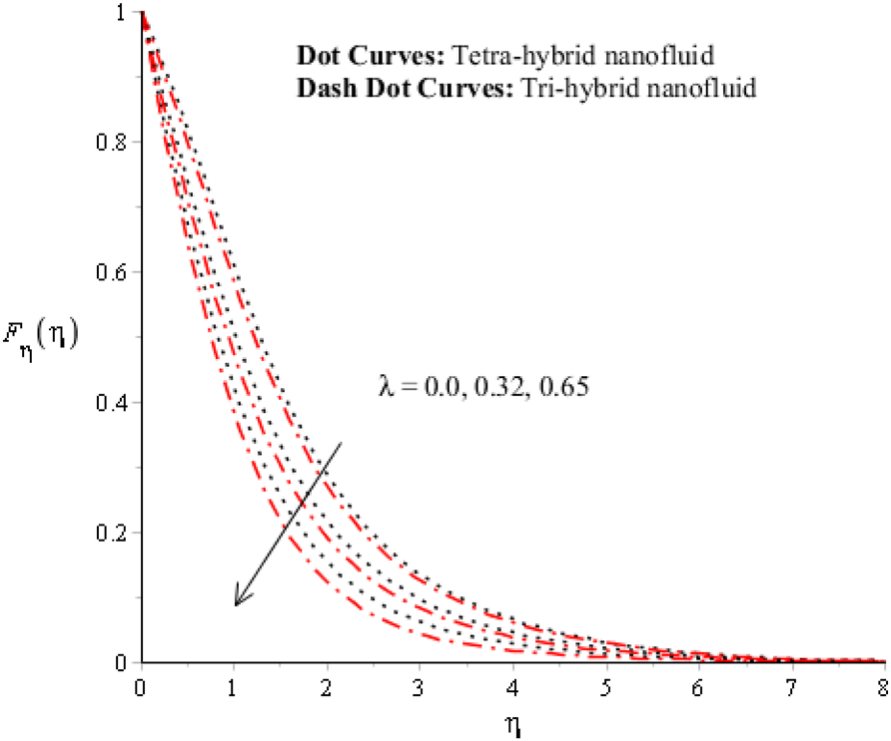
Impression of $\lambda$ on ${F}_{\eta }\left(\eta \right).$

**Figure 8:**
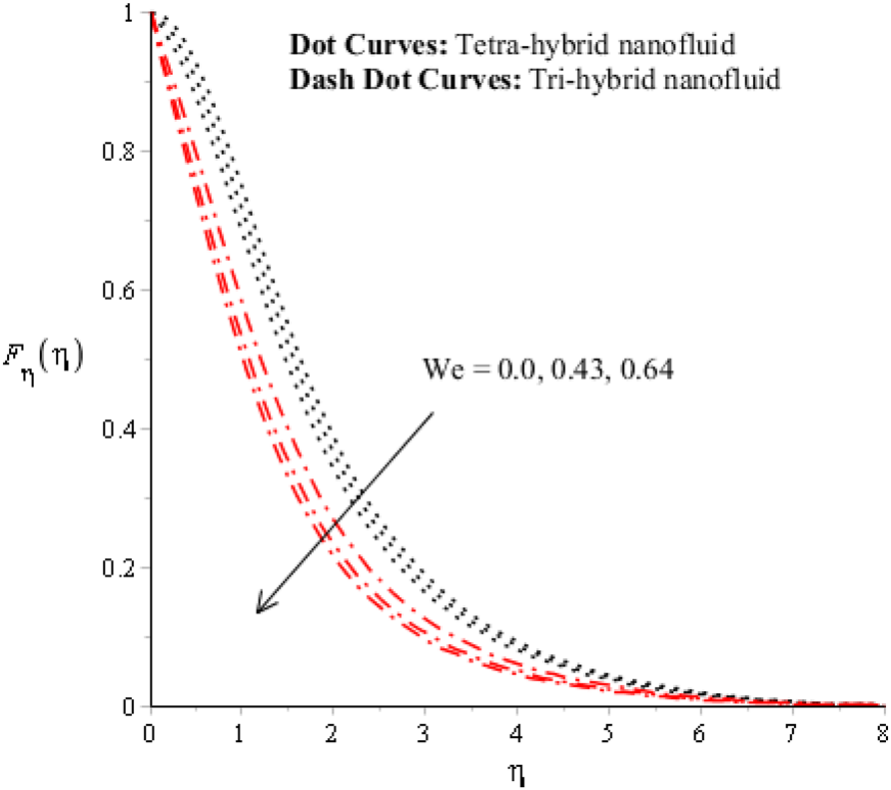
Impression of We on ${F}_{\eta }\left(\eta \right).$

Figures 3, 4, 5, 6, 7 and 8 are plotted for determination of impacts of $$ E^{*}  $$, $$\lambda $$ and $$We$$ on seconder velocity ($$F{\prime}$$) and $$G$$ (primary velocity) incorporating tetra-hybrid nanoparticles and ternary hybrid nanoparticles. Here, a composite of aluminum oxide, iron dioxide, copper and titanium dioxide in sodium alginate is known as tetra-hybrid nanoparticles whereas a composite suspension of aluminum oxide, iron dioxide and copper in sodium alginate is termed as ternary hybrid nanoparticles. Moreover, point dot curves are plotted for interpolation of tetra-hybrid nanoparticles while tri-hybrid nanoparticles are due to dash dot curves. Here, Figs. 3 and 6 are generated for the appearance of solar thermal radiation number ($$ E^{*}  $$) on primary ($$F{\prime}$$) and secondary ($$G$$) velocities. Here, it was interpolated that velocities increase versus enhancement of $$ E^{*}  .$$ $$ E^{*}  $$ is a dimensionless parameter that occurs due to an electric field. Due to the electric field, the temperature of fluids particles increases. Hence, this inclination regarding the temperature of fluids particles results in flow increases. Mathematically, the electric field is directly proportional to velocity field. An enhancement in electric field deals. The velocity field for the case of $$ E^{*}  =0$$ is less than velocity field. When the strength of the electric field is enhanced, it provides a stronger force on fluidic particles and charged particles on the surface of the disk. Such a kind of force can be experienced by the charged particles resulting in accelerated charge particles. Hence, when the electric field is enhanced. It is considered that the charge of the particles exists constant, the force acting on the particles can be increased proportionally. The acceleration of particles is enhanced according to Newton's second law. Figures 4 and 7 show the impression of $$\lambda $$ on $$G(\eta )$$ and $$F{\prime}(\eta )$$ including a mixture of tri-hybrid nanoparticles and tetra-hybrid nanoparticles. Here, $$\lambda $$ is reciprocal magnetic Prandtl and dimensionless numbers. These figures indicate that a rise in $$\lambda $$ inclines $$G(\eta )$$ and $$F{\prime}(\eta )$$ because of significant dominant induction effects rather than magnetic diffusion. Therefore, flow rates are inclined. Physically, this impact is based on frictional forces and the thickness and width of momentum layers are declined. The velocity field is a declining function concerning magnetic Prandtl that is because of enhancing magnetic diffusivity using high values of magnetic Prandtl number. It is influenced that of $$\lambda $$ on velocity field is more prominent at the interface. A little change has been revealed at the stretching disk because of the no-slip boundary condition. When $$\lambda $$ is enhanced, it reveals that magnetic diffusivity becomes less rather than the kinematic viscosity. The velocity field is decreased when $$\lambda $$ is enhanced. Figures 5 and 8 interpolate consequences of $$We$$ on secondary and primary velocities in the tri-hybrid nanoparticles and tetra-hybrid nanomaterials. Physically, this declination regarding the motion of nanoparticles is based on viscous and inertial and viscous forces. Because viscous forces increase when $$We$$ is enhanced. $$We$$ is a dimensionless number that is utilized in the field of viscoelastic fluids. It describes the ratio of the relaxation time and characteristic. Weissenberg number describes the role of viscoelastic materials during flow situations. It is noticed that material takes longer relaxation time as related to characteristic flow. It changes flow behavior when the Weissenberg parameter is increased. The ratio between viscous force and elastic force called as Weissenberg number. An increase in Weissenberg number deals declination of viscous force. Hence, an inclination viscous force deals declination of velocity field. A declination in momentum boundary layers can be noticed when Weissenberg number is magnified.

**Figure 9:**
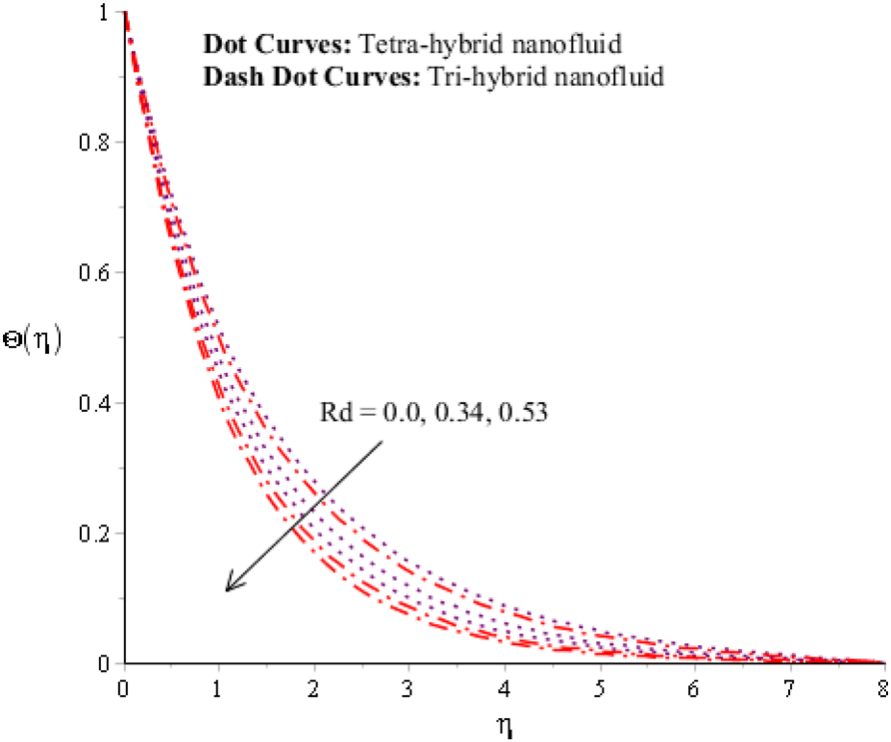
Impression of $Rd$ on $\Theta \left(\eta \right).$

**Figure 10:**
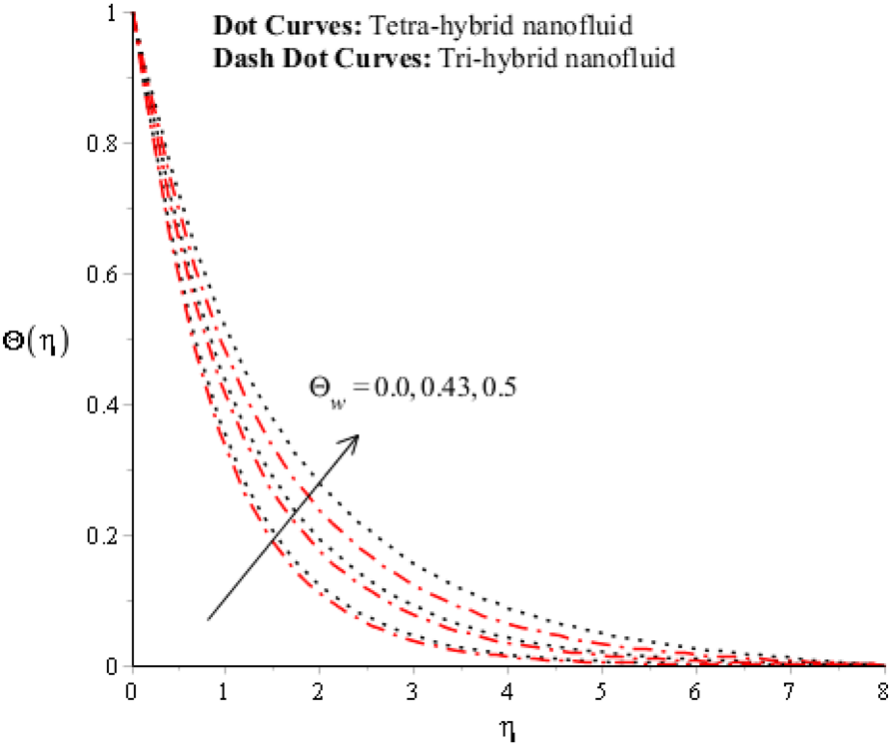
Impression of ${\Theta }_{w}$ on $\Theta \left(\eta \right).$

**Figure 11:**
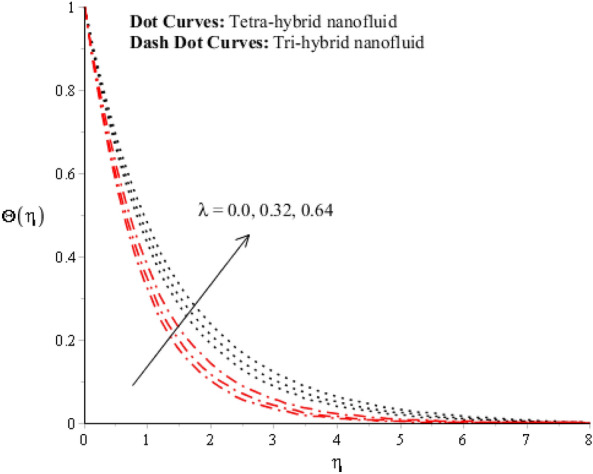
Impression of $\lambda$ on $\Theta \left(\eta \right).$

**Figure 12:**
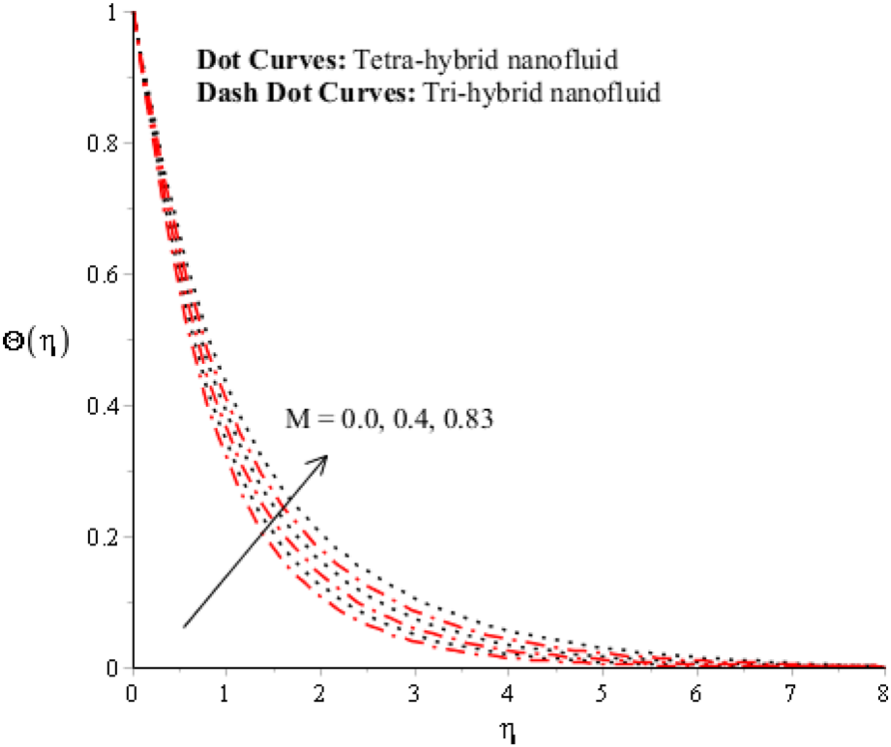
Impression of $M$ on $\Theta \left(\eta \right).$

**Figure 13:**
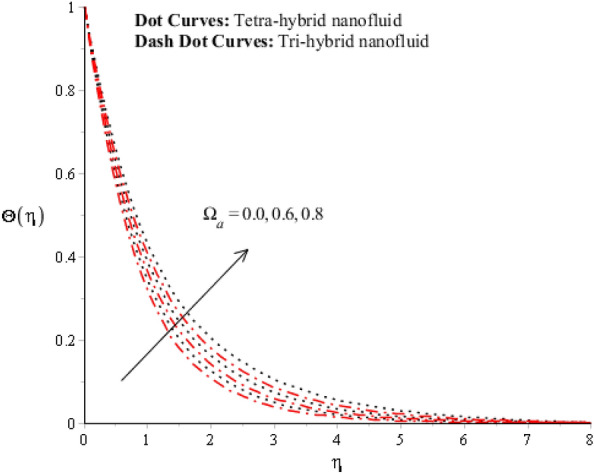
Impression of ${\Omega }_{a}$ on $\Theta \left(\eta \right).$

Figures 9, 10, 11, 12 and 13 reveal thermal features against change in $Rd, {\Theta }_{w}$, $\lambda$, $M$ and ${\Omega }_{a}$. The role of $Rd$ on the temperature profile is addressed in Fig. 9. Temperature profile declines against higher numerical values of $Rd$. Temperature declines based on the concept of thermal radiation. Because transmission of thermal energy transfers using a source of thermal radiation. Hence, radiation along with thermal radiation moves away from the surface. Therefore, heat energy declines when $Rd$ is enhanced. In this figure magnitude of curves for ${Al}_{2}{O}_{3}$-${Fe}_{3}{O}_{4}$-$Cu$-$Ti{O}_{2}$/$SA$ is greater than curves magnitude for ${Al}_{2}{O}_{3}$-${Fe}_{3}{O}_{4}$ -$Ti{O}_{2}$/$SA$. This means maximum production for ${Al}_{2}{O}_{3}$-${Fe}_{3}{O}_{4}$-$Cu$-$Ti{O}_{2}$/$SA$ is greater than thermal production for ${Al}_{2}{O}_{3}$-${Fe}_{3}{O}_{4}$-$Cu$ /$SA.$ Temperature decreases when $Rd$ is enhanced. Plasma exhibits a radiative nature. It radiates heat when it moves. This emission of radiation given electromagnetic waves takes energy away from the fluid. Hence, its temperature decreases. This fact is well supported by the present simulated results. Moreover, the thermal boundary layer thickness declines when $Rd$ is increased. It is investigated to control the thermal boundary layer thickness and radiative plasma can be a more appropriate fluid than usual fluid. Figure 10 interpolates the role of ${\Theta }_{w}$ on the temperature profile. Width and temperature regarding curves increase when ${\Theta }_{w}$ is enhanced. Here, ${\Theta }_{w}$ is formulated using the concept of variable thermal radiation. It was captured that quadratic thermal radiation has been utilized for the formulation of ${\Theta }_{w}.$ Mathematically, a proportional relationship has been observed between ${\Theta }_{w}$ and temperature curves. So, an inclination of ${\Theta }_{w}$ results in temperature increases. ${\Theta }_{w}$ is a dimensionless parameter that is called the temperature difference number in the dimensionless energy equation. By increasing the values of ${\Theta }_{w},$ the temperature difference increases. Hence, an inclination of temperature difference deals with temperature at wall inclines. Moreover, ${\Theta }_{w}$ occurs using the concept of variable thermal radiation in the energy equation. In Fig. 10, the thermal energy for ${Al}_{2}{O}_{3}$-${Fe}_{3}{O}_{4}$-$Cu$ /$SA$ is less than the thermal energy for ${Al}_{2}{O}_{3}$-${Fe}_{3}{O}_{4}$-$Cu$ /$SA.$ Fig. 11 indicates the visualization of $\lambda$ on the thermal profile. Here, $\lambda$ is reciprocal magnetic Prandtl and dimensionless numbers while thermal energy inclines when $\lambda$ is magnified. Physical reasons are based on low magnetic induction and small flow rates versus a large magnitude of $\lambda .$

**Figure 14:**
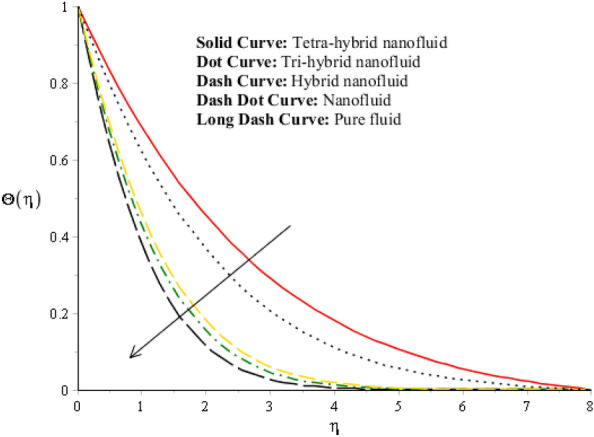
Comparative analysis between tetra-hybrid nano-structures, tri-hybrid nano-martial, nanofluid and pure fluid.

**Figure 15:**
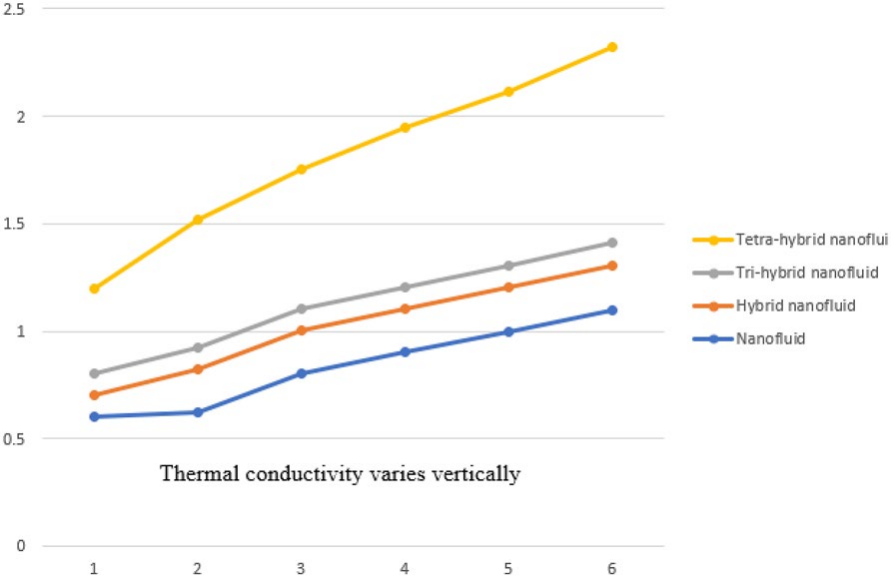
Comparison of thermal conductivity for nano-fluid to tera-hybrid nano-fluid.

**Figure 16:**
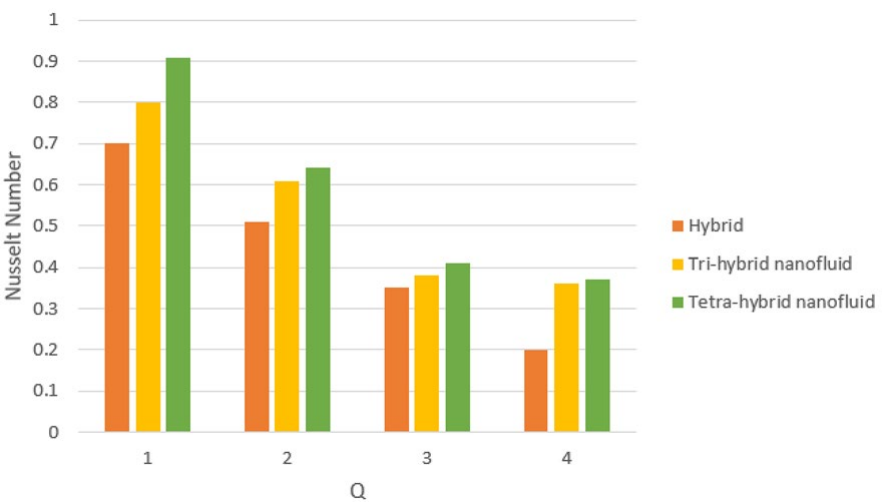
Impression of $Q$ on $Nu.$

**Figure 17:**
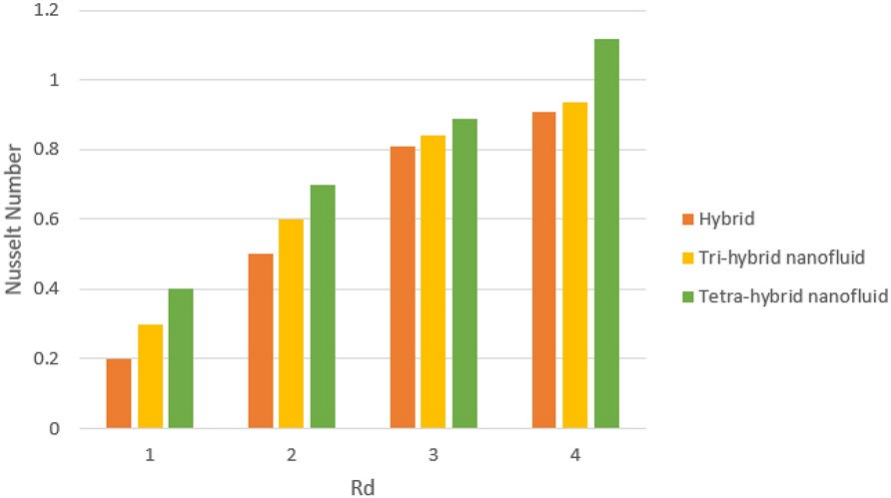
Impression of $Rd$ on $Nu.$

**Figure 18:**
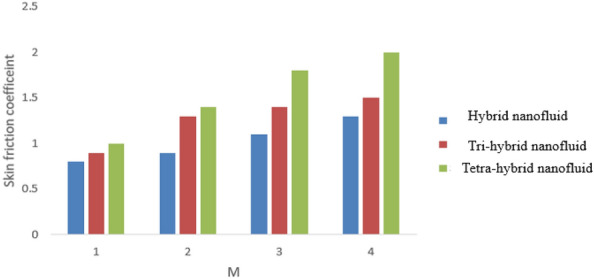
Impression $M$ on the Skin friction coefficient.

A dimensionless parameter ($\lambda$) is a dimensionless parameter that is utilized in the magneto-hydrodynamics field. It is the ratio between magnetic diffusivity and momentum diffusivity multiplied by $Pr.$ An inverse proportional relation between $\lambda$ and thermal diffusivity has been investigated. Hence, an increase in thermal diffusivity deals with maximum enhancement is investigated in thermal energy. Figure 12 estimates the role of magnetic number on temperature profile in the presence of tetra-hybrid nanoparticles and ternary nanoparticles. It was addressed that $M$ (magnetic number) is a dimensionless number. It was determined that the magnetic field is directly proportional to higher intensity related to the magnetic field. Dominating heat dissipation occurs when the process of Joule heating happens. Temperature field enhances. Furthermore, the temperature curve for the case of the hydrodynamic process is less than the thermal energy for the case of the magnetohydrodynamic process. The term associated with the magnetic number occurs in the Joule heating term (in the energy equation). Therefore, a direct relation has been noticed between temperature and magnetic number. Hence, an enhancement of the magnetic field deals enhancement of the temperature field. The formulation of magnetic number is based on the occurrence of Joule heating. An impression of ${\Omega }_{a}$ on the temperature profile is captured in Fig. 13. The formulation of ${\Omega }_{a}$ is based on the non-Fourier's approach while ${\Omega }_{a}$ is the time relaxation number. Thermal energy inclines with various values of ${\Omega }_{a}.$ The parameter associated with ${\Omega }_{a}$ is known as the thermal relaxation number that reveals such kind of property to reestablish its original thermal state. It is established that the magnifying parameter related to ${\Omega }_{a}$ deals rise in temperature. Figures 14, 15 and 16 are plotted characterizations of magnetic number, radiation number and $Q$ on temperature gradient. These figures reveal comparative visualizations among tri-hybrid nanoparticles, tetra-hybrid nanofluid and hybrid nanofluid. The temperature gradient for tetra-hybrid nanofluid is higher than the temperature gradient for nanofluid, tri-hybrid nanoparticles and hybrid nanofluid. Thermal rate (Nusselt number) decreases with large values of $Q$ and Nusselt number inclines versus enhancement of $Rd.$ The flow rate of $M$ is addressed in Fig. 16. In Fig. 16, the flow rate inclines. Tables 3 and 4 are prepared for the estimation of $Rd, We, {\Theta }_{w}$ and $M$ on temperature gradient for ${Al}_{2}{O}_{3}$-${Fe}_{3}{O}_{4}$-$Cu$-$Ti{O}_{2}$/$SA$ and ${Al}_{2}{O}_{3}$-${Fe}_{3}{O}_{4}$-$Cu$/$SA.$ Temperature gradient increases when $Rd$ is enhanced but the opposite role is visualized versus higher numerical values $M, {\Theta }_{w}$ and $We.$ It was essentially estimated that the highest thermal energy for ${Al}_{2}{O}_{3}$-${Fe}_{3}{O}_{4}$-$Cu$-$Ti{O}_{2}$/$SA$ can be achieved as compared to ${Al}_{2}{O}_{3}$-${Fe}_{3}{O}_{4}$-$Cu$/$SA.$ Fig. 17 illustrates a comparison between nanofluid, tri-hybrid nanofluid, hybrid nanofluid and tera-hybrid nanofluid and working fluid. It was observed that maximum heat transfer occurred for the case of tera-hybrid nanofluid rather than for the case of tri-hybrid nanofluid, nanofluid and hybrid nanofluid. Comparative analysis of thermal conductivity between tera-hybrid nanofluid, hybrid nano-fluid, nano-fluid and tri-hybrid nano-fluid is carried out in Fig. 18. It is observed that a homogeneous mixture of ${Al}_{2}{O}_{3}$, ${Fe}_{3}{O}_{4}, Cu$ and $Ti{O}_{2}$ is called a tera-hybrid nano-fluid, homogeneous mixture of ${Al}_{2}{O}_{3}$, ${Fe}_{3}{O}_{4}$ and $Ti{O}_{2}$ is called tri-hybrid nano-fluid, ${Al}_{2}{O}_{3}$ and ${Fe}_{3}{O}_{4}$ is known as hybrid nanofluid. From Fig. 18, thermal conductivity for the case tera-hybrid nano-fluid is higher than thermal for the case tri-hybrid nano-structure, nano-fluid and working fluid.

**Table 3:** Validation of present work in view Skin friction coefficient considering $We=0, \lambda =5.0, {\ell}_{4}=0, {\ell}_{3}=0$.

$\beta$	Published work et al. ^41^	Present work
0.0	0.003467	0.00340103271
0.5	0.002900	0.00299004105
1.0	0.002331	0.00233702176

**Table 4:** Analysis of Nusselt number versus among ${Al}_{2}{O}_{3}$-${Fe}_{3}{O}_{4}$-$Cu$ and ${Al}_{2}{O}_{3}$-${Fe}_{3}{O}_{4}$-$Cu$-$Ti{O}_{2}$ is SA (Sodium alginate) nanofluid.

		${Al}_{2}{O}_{3}$-${Fe}_{3}{O}_{4}$-$Cu/SA$	${Al}_{2}{O}_{3}$-${Fe}_{3}{O}_{4}$-$Cu$-$Ti{O}_{2}$/$SA$
		${({R}_{e})}^{-\frac{1}{2}}Nu$	${({R}_{e})}^{-\frac{1}{2}}Nu$
	0.3	0.2102962137	3.406179061
$Rd$	0.7	0.2259768724	3.510351030
	1.3	0.2310034914	3.608395582
	0.0	0.2359430736	3.442760798
$M$	0.73	0.2107951471	3.377297910
	1.76	0.2056134543	3.357148978
	0.0	0.2503024572	3.5889728027
${\theta }_{w}$	0.54	0.2349135329	3.4208445964
	1.6	0.2194495321	3.1527662019
	0.0	0.2683083958	3.2847390482
$We$	0.75	0.2726352084	3.4167667203
	1.4	0.2768959380	3.6809879610

Table 4 describes the behaviors of tri-hybrid nanoparticles (${Al}_{2}{O}_{3}$, ${Fe}_{3}{O}_{4} and Cu$) on Nusselt number against variation of ($We$) Weissenberg number, ($Rd$) thermal radiation number, ($M$) magnetic field and temperature difference (${\Theta }_{w}$). From Table 4, it is observed that the heat transfer rate for the case of ${Al}_{2}{O}_{3}$-${Fe}_{3}{O}_{4}$-$Cu$-$Ti{O}_{2}$/SA is higher than heat transfer rate for the case of ${Al}_{2}{O}_{3}$-${Fe}_{3}{O}_{4}$-$Cu$ /SA. The heat transfer rate (Nusselt number) declines when magnetic number and temperature difference number are enhanced but the heat transfer rate (Nusselt number) is enhanced when Weissenberg number and radiation number. The behavior of bricks and platelet nanoparticles on the performance of Nusselt number is estimated in Table 5 with the change of ($We$) Weissenberg number, ($Rd$) thermal radiation number, ($M$) magnetic field and temperature difference (${\Theta }_{w}$). From Table 5, it was observed that Nusselt number for the case of bricks nanoparticles is less than heat transfer for the case of platelet nanoparticles.

**Table 5:** Analysis of Nusselt versus among platelet nanoparticles and bricks nanoparticles in Sodium alginate nanoparticles.

		Platelet nanoparticles	Bricks nanoparticles
		${({R}_{e})}^{-\frac{1}{2}}Nu$	${({R}_{e})}^{-\frac{1}{2}}Nu$
	0.3	0.5132896146	0.08096467979
$Rd$	0.7	0.1407195971	0.07715264068
	1.3	0.1414900444	0.07757089190
	0.0	0.1418748975	0.07778045023
$M$	0.73	0.1422594577	0.07799016963
	1.76	0.1426437352	0.07820037197
	0.0	0.1441785431	0.07841080261
${\theta }_{w}$	0.54	0.1445615487	0.07862141674
	1.6	0.1449443176	0.07883249648
	0.0	0.1453266864	0.07925562516
$We$	0.75	0.1457088382	0.07968036343
	1.4	0.1460908409	0.07989323777

## Consequences, strengths and limitations

Thermal characterizations and motion of ${Al}_{2}{O}_{3}$-${Fe}_{3}{O}_{4}$-$Cu$-$Ti{O}_{2}$/$SA$ and ${Al}_{2}{O}_{3}$-${Fe}_{3}{O}_{4}$-$Cu/SA$ are visualized on vertical disk. The behavior of Cross nanomaterial along with magnetic induction is carried out subjected to non-Fourier's theory. The current study is based on tera-hybrid nanofluid, quadratic thermal radiation, shape effects of nanoparticles, and electro-magneto-hydrodynamic. Transformation procedure from PDEs into ODEs utilizing similarity variables that is impactful for solving problems effectively. Utilization of FEM to solve ODEs to provide detailed outcomes and consequences of several parameters. Graphs and tables effectively communicated of findings. The current investigation highlights findings such as manufacturing processes, cancer treatment, and advancement in heating/cooling technologies. Key outcomes and limitations are listed below.

- Terra-hybrid nanoparticles are recommended better in industrial applications where the highest production of thermal energy;
- An enhancement of thermal production can be achieved utilizing different values of the magnetic parameter, time relaxation number, variable thermal radiation number and magnetic induction number but the opposite trend has been noticed with the effects of radiation number;
- Motion of ${Al}_{2}{O}_{3}$-${Fe}_{3}{O}_{4}$-$Cu$-$Ti{O}_{2}$/$SA$ is higher than motion of ${Al}_{2}{O}_{3}$-${Fe}_{3}{O}_{4}$-$Cu/SA;$
- When $E^{*}$ is enhanced results velocity field is enhanced but inverse behavior is noticed with various values of Weissenberg and magnetic induction numbers;
- The highest heat energy can be achieved for bricks nanoparticles as compared to heat energy for platelet nanoparticles.
- Various applications of the current model are cooling systems, aerospace, computer chips, nuclear reactors, drug delivery systems, cancer treatment, food processing, energy efficiency improvements, nuclear reactors, spacecraft design, thermal energy storage, geothermal systems, thermal barrier coating, hypothermia treatment and solar energy, etc.

The limitations of the current investigation are listed below.

- Simplifications that may have limited the results’ applicability or accuracy. For example, making assumptions about perfect circumstances, ignoring particular variables, or using idealized geometries;
- Draw attention to any computational challenges encountered during the investigation that could prevent these methods from being used in certain situations or on a larger scale due to computational demands;
- Discuss any difficulties that exist between the theoretical conclusions and their practical application, as well as any problem that might occur when putting these conclusions to use in actual situations.

## Data Availability

The datasets used and/or analyzed during the current study are available from the corresponding author upon reasonable request.

## List of symbols

$$U, W$$: Velocity components
$H$: Induced magnetic field
$$\pi $$: Phi
$$\sigma $$: Electrical conductivity
$$\nu $$: Kinematic viscosity
$E$: Electric field number
$$k$$: Thermal conductivity
${\sigma }^{*}$: Fluid electrical conductivity
$${T}_{\infty }$$: Ambient temperature
$$a$$: Constant
$${\mathcal{l}}_{3}, {\mathcal{l}}_{2}, {\mathcal{l}}_{1}, {\mathcal{l}}_{4}$$: Volume fractions
$$s1, s2, s3, s4$$: Solid nanoparticles
$tri$: Tri-hybrid nano-fluid
$$bf$$: Base fluid
$$\beta $$: Magnetic parameter
$M$: Magnetic field parameter
$$Rd$$: Radiation number
$\lambda$: Magnetic Prandtl number
$$\infty $$: Infinity
$${R}_{e}$$: Reynolds number
$$Nu$$: Nusselt number
ODEs: Ordinary differential equations
$$r, Z$$: Space coordinates
$$\ddot{\mu }$$: Dynamic viscosity
$$\rho $$: Density
$$E$$: Electric field
$\Gamma$: Time relaxation number
$n$: Power law number
${\delta }_{*}$: Time relaxation number
${C}_{p}$: Specific heat capacitance
$${T}_{W}$$: Wall temperature
$$\eta $$: Independent variable
PDEs: Partial differential equations
$Tetra$: Tetra-hybrid nanofluid
$$hy$$: Hybrid nanofluid
$$Nf$$: Nanofluid
$$We$$: Weissenberg number
$E^{*}$: Electric field number
$$Pr$$: Prandtl number
${\Theta }_{w}$: Temperature difference number
${\Omega }_{a}$: Time relaxation number
${C}_{f}$: Skin friction coefficient
$${\tau }_{w}$$: Wall stress
$${U}_{w}$$: Wall velocity
$$e$$: Elements
